# Comparative Effects of Mineralocorticoid Receptor Antagonism on Organ Dysfunction in COVID-19-Associated ARDS

**DOI:** 10.3390/biomedicines14030731

**Published:** 2026-03-23

**Authors:** Güleren Yartaş Dumanlı, Olcay Dilken, Oktay Demirkıran, Yalım Dikmen, Hafize Uzun, Omur Tabak

**Affiliations:** 1 Department of Intensive Care, Cerrahpaşa Faculty of Medicine, Istanbul University-Cerrahpaşa, 34098 Istanbul, Türkiye; g.yartasdumanli@iuc.edu.tr (G.Y.D.); olcaydilken@gmail.com (O.D.); odemirkiran@yahoo.com (O.D.); yalim.dikmen@gmail.com (Y.D.); 2 Department of Medical Biochemistry, Faculty of Medicine, Istanbul Atlas University, 34403 Istanbul, Türkiye; huzun59@hotmail.com; 3 Department of Internal Medicine, Kanuni Sultan Suleyman Training and Research Hospital, University of Health Sciences, 34303 Istanbul, Türkiye

**Keywords:** COVID-19, SARS-CoV-2, ARDS, spironolactone, furosemide, electrolyte imbalance, intensive care

## Abstract

**Background/Objectives:** Diuretics are recommended for hemodynamically stable patients with COVID-19-associated acute respiratory distress syndrome (ARDS) who have a positive fluid balance. However, furosemide use may be limited by hypokalemia in this population. We aimed to evaluate the clinical and biochemical effects of spironolactone in critically ill patients with COVID-19-associated ARDS. **Methods:** In this retrospective cohort study, 60 patients with COVID-19-associated ARDS admitted to the intensive care unit (ICU) between March and May 2020 were grouped according to diuretic therapy (furosemide vs. spironolactone). Patients were followed for five days (T0–T4). Demographic characteristics and clinical/laboratory parameters were recorded. A two-sided *p* value < 0.05 was considered statistically significant. **Results:** Thirty-one patients received furosemide (F group) and 29 received spironolactone (S group). On day 5, in the F group, cumulative fluid balance and serum sodium increased significantly over time (*p* < 0.05). Lactate increased significantly over time in both groups (*p* < 0.05). N-terminal pro-B-type natriuretic peptide (NT-proBNP) levels increased significantly from T0 to T4 in the F group (*p* < 0.05). **Conclusions:** Spironolactone use was associated with a more favorable trajectory of organ dysfunction and improved volume, electrolyte, and cardiac stress marker dynamics compared with furosemide in patients with COVID-19-associated ARDS. Although confirmation in larger prospective studies is needed, spironolactone may be considered a reasonable diuretic alternative in selected patients, particularly when potassium preservation and avoidance of hypernatremia are clinical priorities.

## 1. Introduction

Since its emergence in late 2019, the coronavirus disease 2019 (COVID-19) pandemic caused by SARS-CoV-2 has posed major challenges in critical care [1,2]. A major cause of mortality in intensive care units (ICUs) is the development of acute respiratory distress syndrome (ARDS), characterized by severe pulmonary inflammation and radiological findings such as ground-glass opacities [3,4].

ARDS is a severe form of acute hypoxemic respiratory failure characterized by diffuse alveolar damage, disruption of the alveolar–capillary barrier, and increased pulmonary vascular permeability. These processes lead to the accumulation of protein-rich fluid in the alveolar space, resulting in pulmonary edema and impaired gas exchange. ARDS is also associated with a pronounced inflammatory cytokine response that contributes to endothelial and epithelial injury and worsens lung dysfunction. Radiologically, ARDS typically presents with bilateral pulmonary infiltrates and ground-glass opacities, while physiologically it is characterized by reduced lung compliance and ventilation–perfusion mismatch, ultimately leading to severe hypoxemia.

In patients with COVID-19, viral infection and dysregulation of the renin–angiotensin–aldosterone system (RAAS) may further exacerbate pulmonary inflammation, endothelial injury, and fluid imbalance. These mechanisms highlight the importance of appropriate fluid management strategies in critically ill patients with ARDS. Recent studies have emphasized the complex inflammatory and pulmonary mechanisms underlying severe respiratory failure and their implications for therapeutic interventions [5,6,7]. In this context, the choice of diuretic therapy may influence both electrolyte homeostasis and pulmonary fluid balance, potentially affecting clinical outcomes in patients with COVID-19-associated ARDS.

The pathogenesis of SARS-CoV-2 is intrinsically linked to the renin–angiotensin–aldosterone system (RAAS). The virus uses the membrane-bound angiotensin-converting enzyme 2 (ACE2) as its primary entry receptor [8,9]. This binding leads to downregulation of ACE2, disrupting the homeostatic balance between the RAAS activator arm (ACE/angiotensin II/AT1R) and its inhibitor arm (ACE2/angiotensin 1–7/MasR) [8,10]. The resulting excess of angiotensin II (ATII) and aldosterone promotes sodium and water retention, potassium depletion, and a pro-inflammatory cascade that increases alveolar–capillary permeability, ultimately contributing to the development of ARDS [8,11,12].

Fluid management remains a cornerstone of ARDS therapy [10,13]. While loop diuretics are often utilized to improve oxygenation in volume-overloaded patients, they may exacerbate the hypokalemia frequently observed in COVID-19 [14,15,16,17]. Consequently, potassium-sparing diuretics, particularly spironolactone, have emerged as a potentially superior alternative [18]. Spironolactone provides a dual therapeutic mechanism: mineralocorticoid receptor (MR) antagonism, which may reduce pulmonary fibrosis and fluid overload, and antiandrogenic effects that may interfere with viral entry [4,19,20,21]. Although it has been proposed as a potential alternative or adjunct to ACE inhibitors and ARBs, clinical data remain scarce [10,22].

This study aimed to determine whether spironolactone provides clinical and biochemical advantages over furosemide in ICU patients with COVID-19-associated ARDS by assessing five-day changes in organ dysfunction, volume status, electrolyte handling, and cardiorenal stress markers, as well as key safety outcomes. We hypothesized that, in the setting of COVID-19–related RAAS dysregulation, spironolactone would attenuate the progression of organ dysfunction, improve sodium–potassium handling (reflected by a higher uNa^+^/uK^+^ ratio with better potassium preservation and reduced hypernatremia), and limit increases in N-terminal pro-B-type natriuretic peptide (NT-proBNP) without compromising oxygenation or safety compared with furosemide.

## 2. Methods

### 2.1. Design and Setting

This single-center, retrospective observational cohort study took place at the Sadi Sun ICU of Istanbul University-Cerrahpasa, Cerrahpasa Medical School, from March to May 2020. The study protocol was developed in accordance with the Declaration of Helsinki and approved by the Istanbul University-Cerrahpasa, Cerrahpasa Faculty of Medicine Clinical Researches Ethics Committee (Date: 7 July 2020; Approval No: 83045809-604.01.02).

### 2.2. Participant Recruitment and Selection

The study consisted of 60 adult patients admitted to the ICU for hypoxemic respiratory failure and requiring diuretic therapy due to positive fluid balance. The COVID-19 diagnosis was made with a positive RT-PCR test for SARS-CoV-2. The patients with a predicted survival longer than 24 h and who were in need of treatment via nasal high-flow oxygen (NHFO), non-invasive mechanical ventilation (NIMV), and IMV due to hypoxemic respiratory failure, and who were hemodynamically stable, were included in the study. ARDS was diagnosed according to the Berlin ARDS definition [23].

Patients were divided into two groups based on the diuretic used according to initial serum sodium and potassium concentrations. spironolactone was administered enterally (2 × 100 mg/day) if serum sodium and potassium concentrations were >140 mEq/L and <3.5 mEq/L, respectively. Intravenous furosemide was administered to the patients without electrolyte abnormalities based upon the clinician’s decision. The exclusion criteria consisted of an age younger than 18 years, chronic renal failure, and routine diuretics use due to congestive heart disease. The first day of the study was named as T0, the following days as T1, T2, T3, and the last day as T4. In order to confirm the absorption and sufficiency of spironolactone given by the enteral route, sodium and potassium levels in spot urine were measured, and the ratio of spot urine sodium to potassium was calculated at the beginning and end of the treatment.

Every patient was provided with the protocolized ARDS treatment, which included restrictive fluid management and a standardized invasive mechanical ventilation management policy, i.e., low tidal volume, positive end-expiratory pressure (PEEP) titration, plateau pressure < 30 cmH_2_O, prone position, and neuromuscular blockers. The diuretic therapy was continued as long as the need persisted during the five days. Continuous renal replacement therapy started in cases without response to diuretic therapy or when uremic complications developed.

### 2.3. Clinical Evaluation

The demographic information (gender and age) and the chronology of events (days from diagnosis to ICU admission and length of ICU stay) were obtained. APACHE II, the Charlson comorbidity index, and SOFA scores were assessed [23,24]. The ventilation status (non-intubated or IMV), vasopressor administration, and the outcome (discharge from ICU or death) were noted.

The oxygenation of patients was calculated by PaO_2_/FiO_2_ formula using the lowest arterial PaO_2_, as previously described [23]. The fluid balance was assessed daily during the study period. Serum concentrations of sodium, potassium, creatinine, blood urea nitrogen (BUN), lactate, and NT-proBNP were measured.

### 2.4. Outcome Measures

The primary outcome was determined as the change in oxygenation (PaO_2_/FiO_2_ ratio), while the secondary outcomes were determined as changes in the SOFA score and mortality. During the study period, patients received standard intensive care management according to institutional protocols, including supportive treatments such as corticosteroids, vasopressors, antiviral therapies, and other medications when clinically indicated. However, due to the retrospective design, potential differences in concomitant treatments between groups cannot be completely excluded. Therefore, the possible influence of these therapies on clinical outcomes should be considered when interpreting the results.

### 2.5. Data Analysis

Descriptive statistics for continuous variables were expressed as mean ± standard deviation (SD) or median (range), as appropriate. Categorical variables were presented as frequencies and percentages. The normality of data distribution was assessed using the Kolmogorov–Smirnov test. Inter-group comparisons for numerical variables were performed using the independent samples *t*-test or Mann–Whitney U test, while categorical variables were analyzed using the Pearson’s chi-square or Fisher’s exact tests. Statistical analyses were interpreted considering baseline sodium imbalance, and results related to sodium changes were evaluated cautiously in the context of potential baseline differences.

Spearman’s rho correlation coefficient was employed to evaluate the relationship between chronological variables and clinical data. Longitudinal changes in numerical variables were analyzed using the Wilcoxon and Friedman tests; where significant differences were identified, the Durbin–Conover post hoc test was utilized to determine the specific time intervals of change. To account for the increased risk of Type 1 error associated with multiple comparisons across various time points and parameters, a Bonferroni correction was applied. Survival outcomes were evaluated using Kaplan–Meier survival analysis. A two-tailed *p*-value of less than 0.05 (after adjustment where applicable) was considered statistically significant. Statistical analyses were conducted using Jamovi (v.1.2.22), JASP (v.0.12.2), and MedCalc Statistical Software (v.15.8, MedCalc Software bvba, Ostend, Belgium).

## 3. Results

**Figure 1:**
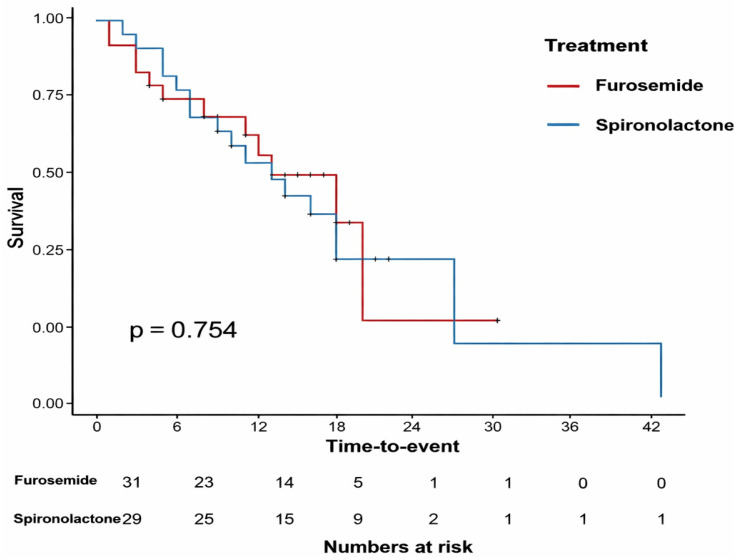
Kaplan–Meier survival curves for patients treated with furosemide and spironolactone.

A total of 60 patients were included. The number of patients in the F and S groups was 31 and 29, respectively. The demographics and clinical data were presented in Table 1. No significant differences in gender and age were present between the groups. The duration from the diagnosis to ICU admission and the length of ICU stay, mean APACHE II score and the Charlson comorbidity index, ventilation strategy, and vasopressor requirement did not differ between the groups. Kaplan–Meier survival analysis demonstrated the time-to-event probability in patients receiving furosemide or spironolactone. Censored observations are indicated by tick marks. The numbers below the graph represent the number of patients at risk at different time points. Survival differences between groups were evaluated using the log-rank test (*p* = 0.754) (Figure 1).

The furosemide dosage was titrated based on individual fluid balance requirements, typically ranging between 40 and 120 mg/day. Statistical analysis confirmed that there was no significant correlation between the furosemide dose and the primary clinical or biochemical outcomes (*p* > 0.05).

No significant differences in PaO2/FiO2 ratio from T0 to T4 and mean SOFA scores from T0 to T3 were present between the groups; however, the mean SOFA score in the F group was significantly increased compared to that of the S group on T4 [7 (1–14) vs. 3 (0–13)] (*p* < 0.05). The fluid balance was similar between the groups, while a significant increase in consecutive days was observed in the F group (*p* < 0.001). Further analysis using the Durbin–Conover test revealed that the significant increases in fluid balance in the F group were present in intervals T0–T2 (*p* = 0.025), T0–T3 (*p* < 0.001), T0–T4 (*p* < 0.001), T1–T3 (*p* = 0.025), T1–T4 (*p* = 0.008), and T2–T4 (*p* = 0.019). While no significant difference in the mean concentration of lactate between the groups was found, a significant increase day by day was observed in each group (*p* = 0.028 and 0.012 for F and S groups, respectively). The mean concentration of NT-proBNP was similar between the groups, while there was a significant increase in the F group from T0 to T4 (*p* < 0.05). No significant differences in the mean concentrations of creatinine and BUN were observed between the groups; however, BUN increased significantly in the S group between T0 and T4 (*p* < 0.05). The mean concentration of serum sodium in the S group on T0 (142 mEq/L) was significantly higher than that of the F group on T0 (137 mEq/L), but on T4, it was significantly lower in the S group (141 mEq/L vs. 148 mEq/L) (*p* < 0.001 and *p* = 0.044, respectively). The mean concentrations of sodium in the F group (*p* < 0.001) significantly increased in intervals T0–T1 (*p* < 0.001), T0–T2 (*p* < 0.001)9, T0–T3 (*p* < 0.001), T0–T4 (*p* < 0.001), T1–T2 (*p* = 0.005), T1–T3 (*p* < 0.001), and T1–T4 (*p* < 0.001). The mean concentration of serum potassium on T2 in the S group (4.6 mEq/L) was significantly higher than of the F group (4.2 mEq/L) (*p* = 0.037). While no change in mean potassium concentrations was observed in the F group, a significant increase in the S group was observed (*p* = 0.011) in intervals T0–T1 (*p* = 0.041), T0–T3 (*p* = 0.002) and T0–T4 (*p* = 0.001). The changes in the clinical and laboratory parameters between the groups and within the groups are shown in Table 2.

Spot urine sodium and potassium ratios (uNa^+^/uK^+^) on day T0 and T4 were evaluated. At T0, uNa^+^/uK^+^ did not differ in both groups (*p* = 0.156). At T4, uNa^+^/uK^+^ was significantly higher in the S group (5.8 vs. 0.7) (*p* < 0.001). Also, both groups were evaluated within themselves, and a statistically significant difference was found between the median values of uNa^+^/uK^+^ at T0 and T4 in the S group (1.2 vs. 5.8) (*p* < 0.001). Accordingly, the uNa^+^/uK^+^ median at T4 in the S group was significantly higher.

The studied variables had been analyzed for any correlations with the duration from diagnosis to ICU admission. A moderate negative correlation was found for PaO2/FiO2 ratios on T0 (r: −0.515; 0:0.004) and T1 (r = −0.413; *p* = 0.026) in the S group. There was also a moderate negative correlation for mean BUN values on T0 in the S group (r = −0.437; *p* = 0.018). In the F group, moderate negative correlations were found for creatinine on T3 (r = −0.451; *p* = 0.021) and NT-proBNP on T4 (r = −0.572; *p* = 0.007). The correlations between variables and the duration between diagnosis and ICU admission were shown in Table 3 and Table 4.

## 4. Discussion

This is the first study showing the beneficial effects of spironolactone in COVID-19 ARDS treated in ICU. The COVID-19 patients who develop ARDS due to increased capillary permeability are highly vulnerable to lung congestion induced by resuscitation. Especially in hemodynamically stable patients, restrictive fluid strategy had been recommended [14]. Neither a significant difference between the groups nor within the groups was observed in PaO_2_/FiO_2_ ratios in the current study. In addition to the fact that proper oxygenation depends on multiple factors besides fluid balance, we suspect that the spironolactone dosage or short course had been insufficient to reverse the oxygenation towards normal limits. Previously, an experimental study reported that spironolactone administration resulted in a 150% increase in the PaO_2_/FiO_2_ ratio in ARDS-induced mice [17,20]. In a retrospective study of Vicenzi et al. [24] canrenone treatment that caused significant improvements in the PaO_2_/FiO_2_ ratio and plasma potassium concentrations had been associated with a positive impact on mortality and clinical improvement in COVID-19 [24]. However, we did not observe any significant difference in the PaO_2_/FiO_2_ ratio between the groups. We suspect that the improvement of oxygenation might be related to RAAS inhibition by the ACEIs and ARBs included in the treatment protocol with canrenone. Moreover, the duration of the study by Vicenzi et al. [24] is different to ours, which might be another factor for the discriminant oxygenation results.

The SOFA score is a good indicator of prognosis in ICU patients [25]. In our study, the mean SOFA scores in both groups did not differ initially. However, on T4, the patients in the F group had significantly higher SOFA scores than the S group. Although the positive predictive value of higher SOFA scores was reported for in-hospital mortality in COVID-19 patients [26], our study is underpowered to detect such a difference. Nevertheless, it is worth noting that a tendency for longer survival in the S group was present (Figure 1). The significant increase in SOFA scores in the F group could be considered as an indicator for a worsening of the general condition of the patients. Although a difference in the SOFA score at day 5 was observed between the treatment groups, no statistically significant difference was detected in the primary outcome measure, the PaO_2_/FiO_2_ ratio. Therefore, the clinical implications of these findings should be interpreted with caution. The observed differences in organ dysfunction scores may represent preliminary signals rather than definitive evidence of treatment efficacy, and larger prospective studies are required to confirm these observations.

Serum lactate levels were determined as a severity marker for COVID-19 in a meta-analysis [27]. Although mean lactate concentrations were similar in both groups, we observed a significant increase in each group from T0 to T4. Interestingly, the actual mean lactate values on each day tended to be lower in the S group compared to that of the F group. Lactate concentration is a marker of tissue hypoxia and was found to be increased in shock status [28]. Increased lactate concentrations have also been associated with higher mortality, and a trend of change in levels was considered valuable for assessing the treatment response and prognosis. The consecutive measurements of lactate levels for longer periods were recommended in critically ill patients [29]. Considering the tendency of decreasing levels of lactate in the S group, we suspect that lactate clearance might have been achieved later if the study would have been continued.

Although no significant difference in fluid balance was observed between the groups, we found a significant increase in the F group throughout the study period. In addition to no significant increase in fluid balance in the S group, we observed a dramatic reduction on T4 without reaching statistical significance; yet it was close (*p* = 0.057). As seen in serum and urine electrolyte analyses, we confirmed the absorption of spironolactone, and even though we observed the positive effects on electrolyte imbalance in 5 days, the steady-state concentrations of spironolactone are usually achieved within 8 to 10 days after initiation of treatment [30]. We can interpret the reduction that started on T4 as the onset time of sufficient spironolactone concentrations for reversing the positive fluid balance.

Low concentrations of serum sodium and potassium were associated with disease severity in COVID-19 [17]. Additionally, a higher incidence of patients presenting with hypernatremia was associated with a longer ICU stay [31]. In this study, serum sodium concentrations of all patients on initial presentation were within normal limits, while the concentrations significantly increased later in the F group, consistent with previous results [31]. Moreover, the mean serum concentration of sodium in the S group (141 mEq/L) was significantly lower than that of the F group (148 mEq/L) on T4 (*p* = 0.044). No abnormal values in potassium concentrations were present in any patients on initial presentation, while a significantly higher value of mean potassium concentration in the S group was observed on T3. Lippi et al. [17] showed that serum sodium and potassium levels were significantly lower in severe COVID-19 in the analyses of five studies with a total sample size of 1415 patients. We suspect that the different initial sodium and potassium concentrations between our study and others might be due to the clinical definitions of studied populations. Our patients were hemodynamically stable and had COVID-19 ARDS, while Lippi defined the patients as “critically ill” or “not” [17]. Additionally, the greater prevalence of hypokalemia in some studies was suggested to be linked with ethnicity-related polymorphisms [18]. It should be noted that baseline serum sodium levels were significantly different between the two treatment groups at T0, with higher median sodium concentrations observed in the spironolactone group. This imbalance may be related to the treatment allocation strategy, which was partially guided by electrolyte levels in clinical practice. Therefore, subsequent differences in sodium levels during follow-up should be interpreted with caution, as they may partly reflect baseline variability rather than a pure treatment-related effect. These findings suggest that the apparent prevention of hypernatremia in the spironolactone group should be considered hypothesis-generating rather than conclusive.

Elevated concentrations of NT-proBNP on admission had been associated with an unfavorable outcome in COVID-19, and it was suggested as an independent risk factor for in-hospital mortality [32]. The NT-proBNP levels were similar between the groups; however, a significant increase in the F group was observed from T0 to T4. This might be interpreted as another piece of evidence for an increased possibility of mortality in F patients.

The levels of BUN and creatinine in COVID-19 patients were found to be higher in fatal cases. Moreover, the researchers concluded that elevated BUN levels were more significantly associated with mortality than increased creatinine levels [33]. We did not detect any significant difference in creatinine and BUN levels between the groups. Considering the positive tendencies in some of the other variables in the S group, we suspect that BUN levels might return to normal given more time.

### Limitations

Several limitations of this study should be acknowledged. First, the relatively short follow-up period may have limited the evaluation of the longitudinal clinical and biochemical effects of spironolactone. Considering that spironolactone and its active metabolites may require approximately 7–10 days to reach steady-state concentrations, the five-day observation period may have captured only partial pharmacological activity. Second, the modest sample size (*n* = 60) may have reduced the statistical power to detect clinically meaningful differences in outcomes such as mortality, oxygenation status (PaO_2_/FiO_2_ ratio), renal function, and progression of organ dysfunction.

Third, the retrospective single-center design may limit the generalizability of the findings and the ability to control for potential confounding variables. Treatment decisions were based on routine clinical practice rather than standardized protocols, and treatment allocation was partly influenced by baseline electrolyte abnormalities, particularly sodium and potassium levels. Consequently, the treatment groups were not fully comparable at baseline, which introduces the possibility of selection bias and confounding by indication. In addition, baseline serum sodium levels differed between groups, and therefore changes in sodium levels during follow-up should be interpreted cautiously.

Another limitation is the heterogeneity in ventilatory support among the study population, which included both non-intubated patients receiving non-invasive respiratory support (NHFO/NIMV) and patients receiving invasive mechanical ventilation. This variability may influence oxygenation indices, fluid balance, and organ dysfunction progression.

Furthermore, direct measurements of renin–angiotensin–aldosterone system (RAAS) biomarkers, including angiotensin II, angiotensin 1–7, aldosterone, and ACE2 activity, were not performed, limiting mechanistic interpretation of the observed findings. Finally, spironolactone was administered enterally in critically ill patients, where gastrointestinal absorption may be unpredictable. Although urinary sodium-to-potassium ratios were evaluated as indirect indicators of pharmacological activity, they cannot fully confirm adequate systemic drug absorption.

Despite these limitations, this study provides real-world clinical data and represents one of the first investigations exploring the potential effects of spironolactone in patients with COVID-19-associated ARDS. However, given the observational design and potential confounding factors, the findings should be interpreted cautiously and considered hypothesis-generating. Larger prospective randomized studies are required to confirm these observations.

The literature on multiple functions of spironolactone, i.e., mineralocorticoid receptor blocking, androgenic inhibition, increasing MasR concentrations, is plentiful, so it would be a narrow perspective to limit its role to diuresis [34]. The hypotheses, as well as the study results, are increasingly pointing towards the importance of RAAS and androgens in the etiopathogenesis of COVID-19 [35,36]. Choosing the appropriate diuretics to add to the restrictive fluid management strategy when needed is especially important in COVID-19 patients with critical electrolyte imbalances, especially hypokalemia [37].

## 5. Conclusions

In mechanically ventilated patients with COVID-19-associated ARDS, spironolactone use was associated with a more favorable trajectory of organ dysfunction compared with furosemide, as reflected by significantly lower SOFA scores on day 5. Although oxygenation indices (PaO_2_/FiO_2_) were similar between groups, the furosemide group demonstrated progressive increases in cumulative fluid balance, serum sodium, and NT-proBNP during the study period. In contrast, spironolactone better preserved potassium levels and was associated with a higher urine sodium-to-potassium ratio on day 5. While no difference in survival was observed, these physiological and organ dysfunction signals suggest that mineralocorticoid receptor antagonism may help mitigate the progression of organ failure in selected patients. However, given the retrospective observational design, short follow-up period, and limited sample size, these findings should be interpreted with caution and considered hypothesis-generating. Larger prospective randomized studies are needed to confirm the potential role of spironolactone in the management of electrolyte balance and fluid homeostasis in patients with COVID-19-associated ARDS.

## Figures and Tables

**Table 1:** Comparison of some demographic and clinical variables according to treatment methods.

	Overall (*n* = 60)	Furosemide (*n* = 31)	Spironolactone (*n* = 29)	*p*
Gender (%)				
Male	43 (71.7)	22 (71.0)	21 (72.4)	0.999 *
Female	17 (28.3)	9 (29.0)	8 (27.6)	
Age (year)	68.0 ± 13.7	67.7 ± 13.7	68.3 ± 13.8	0.874 **
Length of stay in ICU (day)	11.0 [1.0–42.0]	11.0 [1.0–30.0]	13.0 [2.0–42.0]	0.197 ***
Time from diagnosis to ICU admission (day)	3.0 [0.0–70.0]	3.0 [0.0–70.0]	4.0 [0.0–19.0]	0.726 ***
Survival (%)				
Exitus	31 (51.7)	19 (61.3)	12 (41.4)	0.199 *
Alive	29 (48.3)	12 (38.7)	17 (58.6)	
APACHE	16.5 ± 7.0	17.8 ± 7.4	15.1 ± 6.3	0.129 **
Charlson score	4.0 [0.0–11.0]	5.0 [0.0–11.0]	4.0 [0.0–11.0]	0.185 ***
IMV (%)				
T0	31 (51.7)	17 (54.8)	14 (48.3)	0.803 *
T1	29 (49.2)	15 (50.0)	14 (48.3)	0.999 *
T2	28 (48.3)	15 (51.7)	13 (44.8)	0.793 *
T3	32 (58.2)	19 (67.9)	13 (48.1)	0.227 *
T4	28 (56.0)	17 (70.8)	11 (42.3)	0.081 *
Noradrenaline (%)				
T0	21 (35.0)	10 (32.3)	11 (37.9)	0.850 *
T1	22 (38.6)	11 (36.7)	11 (40.7)	0.966 *
T2	21 (37.5)	12 (44.4)	9 (31.0)	0.448 *
T3	16 (30.8)	11 (42.3)	5 (19.2)	0.133 *
T4	18 (39.1)	10 (50.0)	8 (30.8)	0.308 *

*, chi-square test; **, independent *t* test; ***, Mann–Whitney U test.

**Table 2:** Evaluation of selected clinical parameters within and between treatment groups.

	Furosemide (*n* = 31)	Spironolactone (*n* = 29)	*p* *
Sequential Organ Failure Assessment Score (SOFA, points)			
T0	4.0 [1.0–11.0]	3.0 [2.0–11.0]	0.489
T1	4.0 [2.0–12.0]	4.0 [1.0–14.0]	0.649
T2	4.0 [0.0–13.0]	3.0 [0.0–12.0]	0.130
T3	4.0 [2.0–11.0]	3.0 [1.0–12.0]	0.143
T4	7.0 [1.0–14.0]	3.0 [0.0–13.0]	0.022
*p* **	0.853	0.392	
Fluid Balance (mL)			
T0	688.0 [−1560.0–6870.0]	626.5 [−3587.0–5743.0]	0.836
T1	1250.0 [−1630.0–7334.0]	1264.0 [−5197.0–4360.0]	0.247
T2	1299.5 [−2882.0–6684.0]	1047.0 [−5524.0–5743.0]	0.122
T3	2187.0 [−2350.0–11,872.0]	2160.0 [−5170.0–6879.0]	0.134
T4	3100.0 [−2730.0–11,200.0]	500.0 [−5391.0–8355.0]	0.057
*p* **	<0.001	0.321	
PaO_2_/FiO_2_ Ratio (PF ratio, mmHg)			
T0	153.0 [52.0–390.0]	150.0 [48.0–267.0]	0.693
T1	162.5 [63.0–368.0]	150.0 [85.0–400.0]	0.905
T2	139.5 [83.0–485.0]	171.0 [75.0–352.0]	0.640
T3	165.0 [71.0–457.0]	183.0 [74.0–490.0]	0.822
T4	152.0 [92.0–370.0]	177.5 [85.0–494.0]	0.387
*p* **	0.095	0.536	
Lactate (mmol/L)			
T0	1.8 [0.6–7.9]	1.5 [0.9–3.4]	0.087
T1	1.4 [0.8–4.6]	1.4 [0.8–3.1]	0.522
T2	1.9 [0.8–3.9]	1.6 [0.8–3.1]	0.237
T3	1.9 [0.9–4.0]	1.7 [0.8–3.6]	0.200
T4	2.1 [1.0–4.6]	1.9 [0.9–3.5]	0.356
*p* **	0.028	0.012	
Sodium (mmol/L)			
T0	137.0 [125.0–139.0]	142.0 [140.0–152.0]	<0.001
T1	141.0 [134.0–154.0]	141.0 [133.0–152.0]	0.890
T2	143.0 [128.0–154.0]	140.0 [135.0–153.0]	0.078
T3	141.5 [130.0–157.0]	142.0 [137.0–155.0]	0.938
T4	148.0 [130.0–158.0]	141.0 [137.0–152.0]	0.044
*p* **	<0.001	0.349	
Potassium (mmol/L)			
T0	4.3 [2.8–43.0]	4.1 [2.7–5.3]	0.346
T1	4.3 [3.3–6.8]	4.5 [3.0–5.5]	0.28
T2	4.2 [3.1–5.9]	4.6 [3.6–5.8]	0.037
T3	4.4 [3.5–6.2]	5.0 [3.4–6.3]	0.149
T4	4.6 [3.0–5.9]	4.8 [3.6–5.9]	0.316
*p* **	0.281	0.011	
Blood Urea Nitrogen (BUN, mg/dL)			
T0	31.0 [10.0–71.0]	24.0 [7.0–92.0]	0.311
T1	39.0 [13.0–361.0]	27.0 [6.0–114.0]	0.278
T2	38.0 [9.0–81.0]	35.0 [16.0–114.0]	0.992
T3	38.5 [9.0–104.0]	45.5 [9.0–104.0]	0.456
T4	40.0 [10.0–100.0]	36.0 [12.0–99.0]	0.265
*p* **	0.297	0.020	
Creatinine (mg/dL)			
T0	0.9 [0.5–2.8]	1.0 [0.3–2.2]	0.631
T1	1.1 [0.5–2.2]	0.9 [0.2–2.2]	0.718
T2	1.0 [0.4–2.5]	0.9 [0.5–3.4]	0.771
T3	1.2 [0.4–3.7]	1.1 [0.3–2.8]	0.432
T4	1.3 [0.3–5.3]	1.0 [0.2–3.7]	0.157
*p* **	0.132	0.499	
Pro-B-type Natriuretic Peptide (pro-BNP, pg/mL)			
T0	1146.0 [21.5–35,000.0]	2016.0 [8.8–26,834.0]	0.355
T4	2539.0 [96.2–35,000.0]	2478.5 [27.5–35,000.0]	0.666
*p* ***	0.024	0.300	

Data are presented as median [minimum–maximum]. *p* *: Mann–Whitney U test (between-group comparison); *p* **: Friedman test (within-group comparison across time points); *p* ***: Wilcoxon signed-rank test.

**Table 3:** Furosemide-pairwise comparisons (Durbin–Conover).

			Fluid Balance	Lactate	Sodium
T0	-	T1	0.056	0.765	<0.001
T0	-	T2	0.025	0.044	<0.001
T0	-	T3	<0.001	0.031	<0.001
T0	-	T4	<0.001	0.008	<0.001
T1	-	T2	0.733	0.084	0.005
T1	-	T3	0.025	0.061	<0.001
T1	-	T4	0.008	0.019	<0.001
T2	-	T3	0.056	0.881	0.312
T2	-	T4	0.019	0.518	0.058
T3	-	T4	0.649	0.619	0.362

**Table 4:** Spironolactone-pairwise comparisons (Durbin–Conover).

			Lactate	Potassium	BUN
T0	-	T1	0.708	0.041	0.039
T0	-	T2	0.037	0.102	0.256
T0	-	T3	0.041	0.002	<0.001
T0	-	T4	0.002	0.001	0.051
T1	-	T2	0.085	0.668	0.338
T1	-	T3	0.094	0.224	0.170
T1	-	T4	0.006	0.202	0.904
T2	-	T3	0.963	0.102	0.022
T2	-	T4	0.282	0.090	0.402
T3	-	T4	0.262	0.951	0.137

## Data Availability

The data that support the findings of this study are available on request from the corresponding author. The data are not publicly available due to privacy or ethical restrictions.
